# Atrial Flutter With Intraventricular Conduction Delay, Hypotension, and Bradycardia in the Setting of High-Output Ileostomy With Renal Failure, Hyperkalemia, and Metabolic Acidosis: A Case Report With Brief Literature Review

**DOI:** 10.7759/cureus.84158

**Published:** 2025-05-15

**Authors:** Edinen Asuka, Barbara Odac, Andrew Ndakotsu, Anastasia Postoev

**Affiliations:** 1 Internal Medicine and Preventive Medicine, Griffin Hospital, Derby, USA; 2 Internal Medicine, MedStar Health, Baltimore, USA; 3 Academic Medicine, Caribbean Medical University, Willemstad, CUW

**Keywords:** atrial fibrillation, atrial flutter, bradycardia, end-organ dysfunction, high-output ileostomy, high-output ostomy, hyperkalemia, metabolic acidosis, renal failure, volume depletion

## Abstract

After ostomy creation, some patients tend to develop high outputs from their ostomy site, which puts them at risk for volume depletion, renal failure, and electrolyte and acid-base abnormalities. This case emphasizes the need for a multidisciplinary team-based approach for the care of patients after ostomy creation, especially those with persistent high output and significant cardiac comorbidities, including cardiac arrhythmias. Patient education and subsequent follow-ups and assessments are important to prevent life-threatening complications in these patients.

## Introduction

High-output ostomy is a complication that may occur after the surgical creation of an ileostomy. Some of these patients tend to develop some complications, such as high output from the ostomy site, which can lead to overt volume depletion and acid-base and electrolyte imbalances. In the setting of volume depletion, renal perfusion is decreased, which can precipitate renal failure and impaired potassium and acid excretion in the kidney. On the other hand, high output from the ostomy site may also cause loss of electrolytes, including potassium [1-8]. Patients with atrial flutter or fibrillation are often placed on rate-controlling atrioventricular node-blocking agents or antiarrhythmic medications. It is crucial to understand the interaction between both pathologies and how high output from the ostomy can negatively impact patients with significant cardiac comorbidities, particularly those with underlying cardiac arrhythmias, such as atrial flutter or atrial fibrillation, as most of these patients tend to be on rate-controlling or antiarrhythmic medications [5-8]. When these patients develop high output from their ileostomy site while on the above-mentioned agents, especially in the setting of electrolyte abnormality such as hyperkalemia, it can lead to significant conduction impairment or abnormalities within the heart. Here, we discuss the interplay between these conditions, the need for a multidisciplinary approach to care, patient education, and the need for close follow-up and assessment.

## Case presentation

A 72-year-old male had a medical history of asthma, hypertension, hyperlipidemia (on statin), type 2 diabetes mellitus, heart failure with preserved ejection fraction, paroxysmal atrial fibrillation and flutter (on anticoagulant apixaban), previous opioid and alcohol use disorder (in remission), and diverticulitis (status post-sigmoid colectomy and anastomosis on previous admission), though later noted to have developed tubulovillous adenoma with high-grade dysplasia around the site of colorectal anastomosis, requiring resection with a diverting ileostomy creation to aid proper healing of the colorectal anastomosis. After months of outpatient follow-up, the patient was referred to the emergency department by his surgery specialist due to significantly deranged blood work results after reporting recurrent high output from his ileostomy site and generalized fatigue.

Objective findings

**Figure 1:**
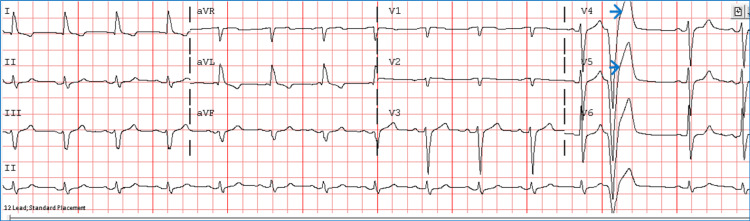
ECG before admission showing sinus rhythm and normal ventricular rate with premature ventricular complexes (blue arrows).

**Figure 2:**
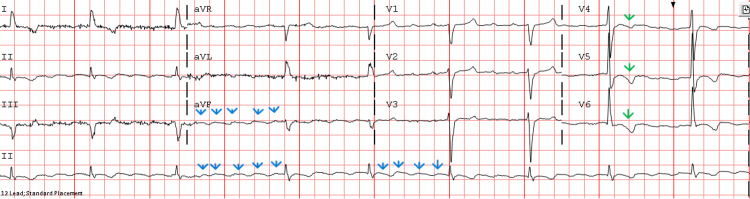
ECG at presentation showing atrial flutter (flutter waves, blues arrows) with intraventricular conduction delay (IVCD), bradycardia, and nonspecific T-wave inversions in V4-V6 (green arrows) in the setting of IVCD.

On presentation, the patient was noted to be hypotensive, with bradycardia at 30-50 beats/minute, and maintaining optimal saturation on room air. Examination findings revealed an acutely ill-looking patient with a weak, thready pulse with S1 and S2 heart sounds, no obvious wheezing or crepitation on auscultation, no overt abdominal distention, no overt abdominal tenderness, and no rebound or guarding. Ostomy appeared functional with effluence in the ostomy bag at the time of evaluation. No extremity edema was observed, and other aspects of the examination were unremarkable. A previous ECG (Figure 1) and ECG at presentation (Figure 2) revealed atrial flutter with intraventricular conduction delay (IVCD), bradycardia, and nonspecific T-wave inversions in V4-V6 in the setting of IVCD. Initial laboratory work (Table 1) revealed hyperkalemia, high anion gap metabolic acidosis, acute renal failure, random blood glucose level within range, normal lactic acid level, and normal high-sensitivity troponin level. His complete blood count revealed no leukocytosis and showed findings of chronic normocytic anemia and mild thrombocytosis. Chest X-ray showed no acute cardiopulmonary findings. Renal ultrasound showed bilateral renal simple cystic foci, 7 mm on the right and 3.3 cm on the left, with no nephrolithiasis or hydronephrosis. A transthoracic echocardiogram (TTE) performed about two weeks before admission revealed a normal ejection fraction of 60%, no wall motion abnormality, no significant valvular abnormality, and a severely dilated left atrium. He was admitted to the intensive care unit for treatment and close monitoring.

**Table 1:** Laboratory results with the respective reference ranges.

Laboratory parameters	Results	Reference range
Potassium	6.6	3.5–5.1 mmol/L
Anion gap	19	5–16 mmol/L
Venous blood gas pH	7.11	7.31–7.41
Arterial blood gas pH	7.23	7.35–7.45
Arterial blood gas pCO2	23.3	35–45 mmHg
Arterial blood gas pO2	107.1	80–100 mmHg
Arterial blood gas bicarbonate	9.5	22–28 mEq/L
Serum creatinine	10.72	0.73–1.18 mg/dL
Random blood glucose	191	<200 mg/dL
Lactic acid level	1.6	0.5–1.9 mmol/L
High-sensitivity troponin I	47.47	Female: <34.0 ng/L, male: <54.0 ng/L
White blood cell count	7,600	4,800–10,800 cells/μL
Hemoglobin level	10.9	14–18 g/dL
Mean corpuscular volume	85.2	80.0–94.0 fL
Platelet count	556,000	140,000–440,000 platelets/μL

Interventions utilized and treatment approach

**Figure 3:**
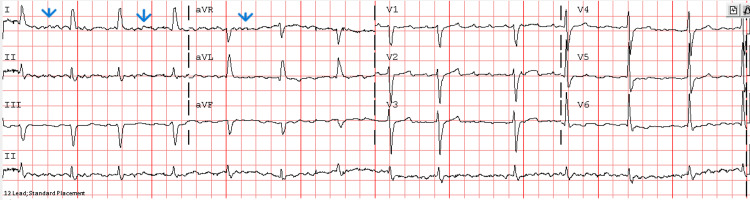
ECG showing atrial flutter with normal ventricular rate (flutter waves, blue arrows) and no significant ST-T abnormality.

The patient received intravenous calcium gluconate, intravenous dextrose 50% with intravenous regular insulin x1 dose, oral sodium zirconium, and intravenous fluid bolus. Thereafter, he was started on sodium bicarbonate infusion, norepinephrine infusion titrated per protocol starting around 5 µg/minute (to maintain mean arterial pressure >65 mmHg), and dopamine infusion at 5 µg/kg/min (later added to achieve target heart rate >60 beats/minute) per protocol. The cardiology, nephrology, and surgery teams were duly consulted for further input. His CHA₂DS₂-VASc score was around 4. The patient had already had his apixaban before presentation, which was later restarted during his hospital stay. ECG after clinical improvement and resolution of electrolyte imbalance, hyperkalemia, metabolic acidosis, and renal function is presented in Figure 3. His routine home antihypertensive medications, such as lisinopril and anti-chronotropic or atrioventricular blocking agents, including metoprolol and diltiazem, were held at presentation. Electrolyte derangements were corrected, including hyperkalemia (likely secondary to existing metabolic acidosis and renal impairment). Likewise, correction of volume depletion and metabolic acidosis was attained as well. He was gradually weaned off norepinephrine and dopamine infusions per protocol as his blood pressure and heart rate normalized. The sodium bicarbonate infusion was later discontinued after the resolution of his metabolic acidosis. Thereafter, he was started on intravenous lactated Ringer’s fluid with close monitoring of ileostomy output, later discontinued as his intake and output, including renal function, markedly improved to his baseline. He was placed on antimotility agents such as diphenoxylate-atropine (2.5/0.025 mg) three times daily, adjusted accordingly to curtail high output from the ostomy site. Of note, he had previously tried loperamide and fiber supplementation, though high output from the ileostomy site persisted or reoccurred despite the above-mentioned intervention. It is also important to note that this was his second admission with similar issues. Once the patient was optimized and acute end-organ dysfunction, electrolyte, and electrophysiologic abnormalities had resolved, a shared decision was made with the patient to perform ileostomy reversal. Confirmation of intact and patent colorectal anastomosis was ensured by the surgery team per protocol including colonoscopy, with functionality of anal sphincter and bowel control confirmed before ileostomy reversal. He was seen by the cardiac electrophysiology team. Given the large left atrial enlargement noted on his recent cardiac echocardiogram, his chance of maintaining sinus rhythm after ablation was deemed low, and with consideration of his acutely ill state while on admission, a shared decision was made with the patient to aim for rate control and reassess the need for ablation therapy on an outpatient basis.

Discharge and follow-up

The patient’s cardiac medications were gradually restarted as tolerated before discharge, including metoprolol and diltiazem, and Eliquis thereafter. Close outpatient follow-up appointments were scheduled with the cardiology and cardiac electrophysiology teams for further titration upon discharge. He was also provided a close outpatient follow-up appointment with the surgery and endocrinology team for further evaluation. Two to three weeks after discharge, the patient was seen by the respective teams on an outpatient basis with no acute issues noted.

## Discussion

Patients with an ileostomy can develop a high output of >1.5 L per day. If not promptly rectified, this can lead to complications such as end-organ dysfunction, metabolic acidosis, and electrolyte abnormalities such as hyperkalemia, which may occur in the setting of acute renal failure, as in this case, or hypokalemia due to loss of electrolytes from the ostomy drainage, both of which can have detrimental effects on the heart [8-11]. Our patient had pertinent cardiac comorbidities, including heart failure with preserved ejection fraction and a history of paroxysmal atrial fibrillation and flutter. When coupled with the persistently high ostomy output, this increased his risk of morbidity and mortality. Oftentimes, patients with an underlying history of atrial flutter or fibrillation tend to be on rate-controlling atrioventricular node-blocking agents or antiarrhythmic medications; hence, persistently high output from ostomy sites, as in this case, predisposes patients to significant electrolyte derangements and acid-base abnormalities, which can be very harmful to these patients. Soluble, viscous, non-fermenting, gel-forming fiber supplementation, such as psyllium husk, is often recommended for patients with high-output ostomies [8-13]. This slows transit time by absorbing water and forming gel-viscous substances. Likewise, adequate hydration is recommended. Therefore, in addition to the daily recommended intake for the general population (2.7-3.7 L), an additional intake of 500-750 mL is often recommended [8-13]. On the other hand, insoluble fiber supplements tend to increase bowel transit time and are primarily used to treat constipation. For patients exhibiting poor response to fiber supplementation, antimotility medications are frequently incorporated to help prevent recurrence or persistence of high output from ostomy sites. Some of these medications include loperamide and diphenoxylate-atropine [8-13]. Likewise, antisecretory agents such as octreotide, proton-pump inhibitors, and histamine-2 antagonists have been shown to be helpful adjuncts. In severe cases, especially when conservative management with the above-mentioned approaches fails, it is often advisable to perform a reversal of the ostomy stoma and restoration of bowel continuity, if possible [10-13]. In this case, surgical reversal was pertinent as our patient was noted to have recurrent life-threatening abnormalities coupled with his underlying cardiac comorbidities.

## Conclusions

This case underscores the need for multidisciplinary team-based care, patient education, and the need for close follow-up of high-risk patients with underlying cardiac comorbidities, such as cardiac arrhythmias, after ostomy creation. Patient education on diet and close outpatient follow-up, especially during the initial phase, is ideal to aid prompt intervention if the need arises. We also discussed possible complications to look out for in patients with significant output from their ostomy sites, such as significant volume depletion, end-organ dysfunction, renal failure, acid-base abnormalities such as metabolic acidosis, as well as life-threatening electrolyte abnormalities, such as hyperkalemia, especially for high-risk patients with cardiac comorbidities, including those on rate-control medications or antiarrhythmics. For recurrent cases, if conservative measures, such as dietary modifications and utilization of antimotility agents, fail, surgical intervention is often recommended, which may entail the reversal of the ostomy stoma, if feasible, to prevent life-threatening complications in these patients.
